# Phase angle values, a good indicator of nutritional status, are associated with median value of hemoglobin rather than hemoglobin variability in hemodialysis patients

**DOI:** 10.1080/0886022X.2020.1870137

**Published:** 2021-02-10

**Authors:** Do Hyoung Kim, Dong-Jin Oh

**Affiliations:** a Division of Nephrology, Department of Internal Medicine, Hallym University Kangnam Sacred Heart Hospital, Seoul, Korea; b Division of Nephrology, Department of Internal Medicine, Myongji Hospital, Hanyang University College of Medicine, Goyang, Korea

**Keywords:** Hemodialysis, hemoglobin, variability, nutrition, phase angle

## Abstract

**Background:**

Our aim was to elucidate whether Hb variability affects nutritional status in HD patients.

**Methods:**

This study included chronic HD patients (*n* = 76) with available monthly Hb levels up to 24 months prior to the body composition monitoring (BCM) measurement. The parameters obtained in the BCM included body mass index (BMI), lean tissue index (LTI), fat tissue index (FTI), body cell mass index (BCMI), overhydration/extracellular water ratio (OH), and phase angle (PhA). The coefficient of variation (Hb-CV), standard deviation (Hb-SD), and range of Hb (Hb-RAN) were used as indexes of Hb variability. In addition, minimum (Hb-Min), maximum (Hb-Max), average (Hb-Avg), and median (Hb-Med) Hb levels (g/dL) were analyzed.

**Results:**

There were no significant differences in clinical, biochemical, and nutritional indexes based on the Hb-CV level. Compared to patients with an Hb-Med ≤ 10.77, those with an Hb-Med >10.77 had higher albumin levels, total iron-binding capacity (TIBC), and PhA and lower average weekly prescribed darbepoetin. Age, female sex, OH, and darbepoetin dosage were negatively correlated with PhA. Serum albumin, phosphorus, TIBC, Hb-Med, and Hb-Avg were positively correlated with PhA. In multiple linear regression analysis, PhA was positively associated with Hb-Med and serum albumin level, whereas PhA was negatively associated with age and female sex. The area under the curve (AUC) of Hb-Med was 0.665 (*p* = 0.040) in predicting PhA >5.00°

**Conclusions:**

PhA was not affected by indexes of Hb variability, whereas PhA was associated with Hb-Med in chronic HD patients.

## Introduction

Anemia is a common complication of patients with chronic kidney disease (CKD) and end-stage renal disease (ESRD) undergoing renal replacement treatment. Although the introduction of erythrocyte-stimulating agents (ESAs) has led to a dramatic reduction in blood transfusion requirements and is associated with improved quality of life, fluctuations in hemoglobin (Hb) levels, known as Hb variability, during ESA treatment is a well-documented phenomenon [1]. The optimal target Hb concentration in CKD/ESRD patients remains under debate. Countries and societies have different recommendations for maintaining the optimal Hb concentration. In addition, maintaining patients’ Hb levels in such an optimal narrow range is difficult due to the loss of physiological regulation of red blood cell formation and many other factors, such as iron deficiency, chronic inflammation, secondary hyperparathyroidism, malnutrition, and inadequate-dose dialysis. The data show that only 30% of patients will fall within this range at any point in time because Hb level fluctuations result in frequent under- and overshooting of the target level [2]. Chronic HD patients with a stable target Hb level are at lower risk for adverse events than maintenance HD patients without a stable Hb level [3]. In recent studies, a higher fluctuation in Hb variability was associated with cardiovascular mortality and all-cause mortality in maintenance HD patients [4,5]. Malnutrition is a well-known risk factor in the general population and in HD patients. However, few studies have evaluated the association between Hb variability and nutritional status in HD patients. The nutritional status in HD patients is usually assessed using the malnutrition-inflammation score (MIS), body mass index (BMI), and serum albumin level. This was because there was a problem with the accurate and reproducible evaluation of nutrition status. Body composite monitor (BCM) analysis is a noninvasive and accurate instrument for distinguishing between excessive and insufficient moisture levels and can accurately assess nutritional status [6,7]. Therefore, we aimed to elucidate whether Hb variability itself affected the nutritional status.

## PATIENTS and METHODS

### Study population

**Figure 1.**
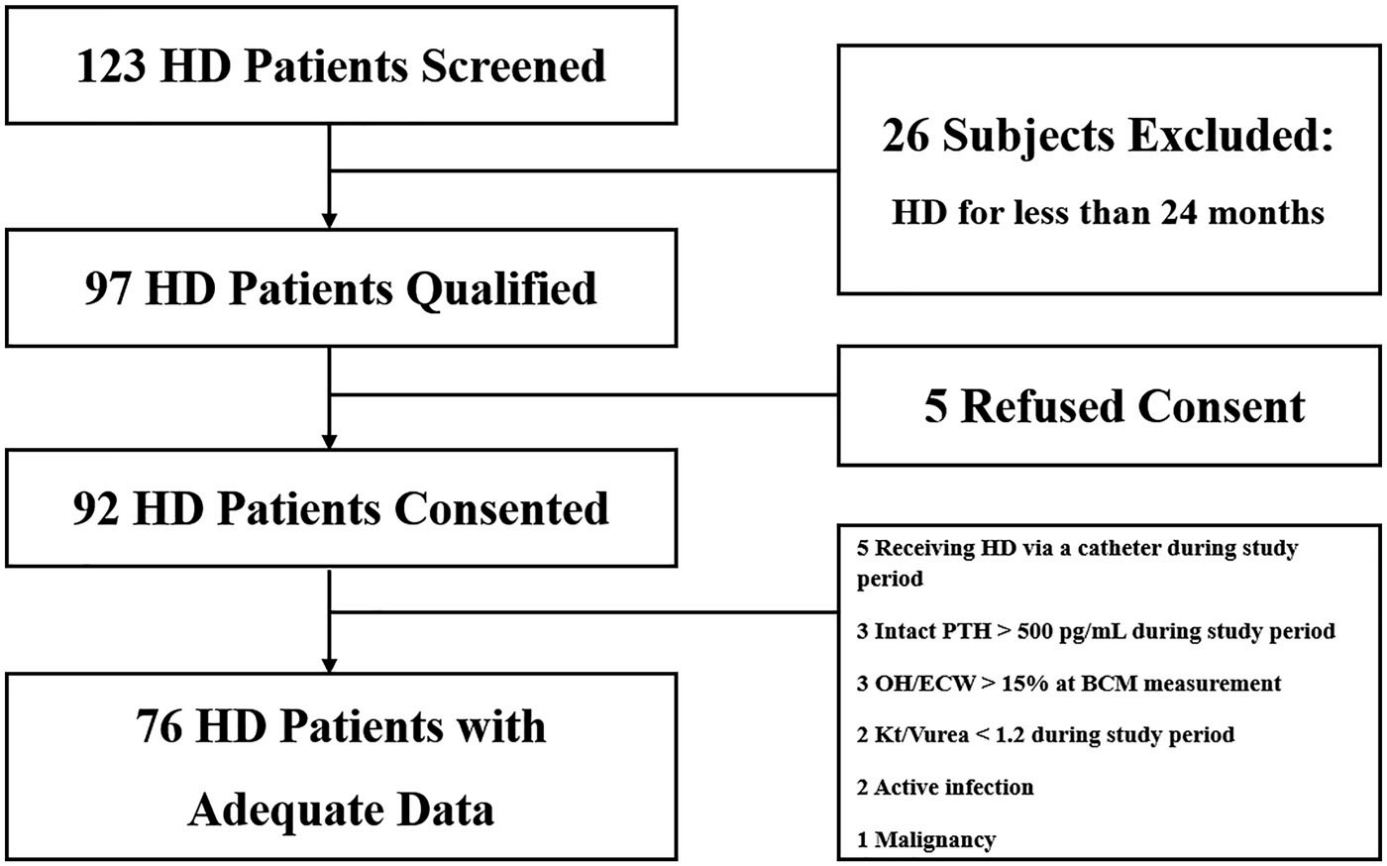
Study Flow Diagram.

Patients with ESRD who were receiving maintenance HD were recruited. This study was conducted between 1 December 2016 and 1 May 2019 and included patients from Myongji Hospital in Korea. The study included adult patients aged over 20 years who had been undergoing HD for more than 24 months (Figure 1). The target Hb level was 10–11 g/dL according to reimbursement regulations of the Korea Health Insurance Review & Assessment Service. Dose adjustments of darbepoetin (NESP®; Kyowa Kirin Korea Co., Ltd., Seoul, Korea) were made according to the Hb level measured monthly in our facility. Therefore, every study population had more than 24 monthly Hb data points. The patients with acute infection, with malignancy, intact PTH > 500 pg/mL during study period, overhydration/extracellular water > 15% (OH) at BCM measurement, Kt/Vurea < 1.2 during study period or who were receiving HD *via* a catheter were excluded. Finally, 76 patients were enrolled in the study. Informed written consent was obtained from all patients. This study was approved by our facility’s institutional review board [MJH2018-10-012].

### Determination of Hb indexes

Hb-CV, Hb-SD, Hb-RAN, Hb-Min, Hb-Max, Hb-Avg, and Hb-Med were the Hb indexes. All the Hb indexes were calculated using monthly Hb levels for at least 24 months prior to the BCM test. We quantified the degree of Hb variability using range (HD-RAN), standard deviation (Hb-SD), and coefficient of variation of Hb (Hb-CV), that is, the ratio of standard deviation to the average Hb level [8]. BCM was performed at the study population enrollment.

### Other data collection

All demographic and clinical data were collected from the patients’ electronic medical records. Age, sex, height, body weight, presence of diabetes, HD duration, and average laboratory data for the previous 24 months before BCM were collected. Laboratory data included mean platelet volume (MPV), total iron-binding capacity (TIBC), transferrin saturation (TS), and levels of serum iron, ferritin, albumin, calcium, phosphorus, intact parathyroid hormone (PTH), triglyceride, total cholesterol, high-density lipoprotein (HDL)-cholesterol, low-density lipoprotein (LDL)-cholesterol, and highly sensitive C-reactive protein (*hs*-CRP). Darbepoetin dosages were collected during the entire study period and calculated as µg/week.

### BCM analysis

The nutritional status of the patients at enrollment was assessed using the BCM^®^ (Fresenius Medical Care a Deutschland GmbH, Germany). The measurements were made before the onset of the dialysis session at mid-week with 4 conventional electrodes being placed on the patient, who was lying in the supine position: 2 on the hand and 2 on the foot contralateral to the vascular access. The parameters obtained with the BCM were BMI, LTI, FTI, BCMI, and PhA [9].

### Statistical analysis

Because of the small number of patients, normal distribution was tested using a single sample Kolmogorov–Smirnov analysis. Variables are expressed as mean ± SD or median (range). Between-group differences were assessed for significance using Mann–Whitney *U* tests. Correlations between nutritional parameters and clinical, biochemical, and Hb indexes were assessed using Spearman’s (non-parametric) correlations. Multivariate linear regression analysis was used to assess the combined influence of the PhA values adjusted for age, sex, TIBC, albumin level, phosphorus level, and all Hb indexes. We evaluated the receiver operating characteristics (ROC) curve of Hb-Med, Hb-CV for predicting PA > 5.00°, which is considered as PhA cutoff value for estimating well-nourished status proposed in previous research [6]. Significant differences were defined as *p* values less than 0.05. All statistical analyses were performed using Statistical Package for the Social Sciences version 23.0 (SPSS Inc., Chicago, IL).

## Results

### Clinical characteristics of the study population

The study included a total of 76 patients undergoing HD (median duration, 70.98 (24–130) months; 51.3% men; mean age, 65.84 ± 11.91 years). Here, 51.6% patients had diabetes mellitus. The mean Hb and darbepoetin dosage were 10.80 ± 0.51 g/dL and 25.08 ± 13.41 µg/week. The mean albumin, iron, TIBC, TS, total cholesterol, LDL-cholesterol, HDL-cholesterol, triglyceride, calcium, phosphorus, intact PTH, and hs-CRP levels were 3.83 ± 0.22 g/dL, 73.78 ± 17.75, 257.20 ± 30.68 µg/dL, 28.81 ± 6.44%, 134.86 ± 26.45, 67.92 ± 20.63, 46.04 ± 13.00, 104.95 ± 47.23, 8.36 ± 0.38, 4.10 ± 0.66, 313.23 ± 159.41, and 0.42 ± 0.43 mg/dL, respectively. The median ferritin was 228.18 (49.14–1233.45). The dry weight, pre-dialysis weight, interdialytic weight gain, and OH were 60.80 ± 10.34, 62.30 ± 11.36, 2.16 ± 0.94 kg, and 8.47 ± 6.52%. The mean BMI, LTI, FTI, BCMI, and PhA were 23.58 ± 3.02, 12.71 ± 3.17, 9.80 ± 3.59, 6.92 ± 2.25 kg/m^2^, and 4.28 ± 0.94°, respectively (Table 1).

**Table 1.** Clinical characteristics of the study population (*N* = 76).

Variable	Value
Age (year)	65.84 ± 11.91
Sex (*n*, %)	
Male	39 (51.3)
Female	37 (48.7)
Dialysis duration (months)	70.98 (24–130)
Diabetes mellitus (%)	40 (52.6)
Dry weight (kg)	60.80 ± 10.34
Pre-dialysis weight (kg)	62.30 ± 11.36
Interdialytic weight gain (kg)	2.16 ± 0.94
OH at pre-dialysis (%)	8.47 ± 6.52
Hemoglobin (g/dL)	10.80 ± 0.51
Mean platelet volume (fL)	10.02 ± 0.75
Albumin (g/dL)	3.83 ± 0.22
Iron (µg/dL)	73.78 ± 17.75
TIBC (µg/dL)	257.20 ± 30.68
Transferrin saturation (%)	28.81 ± 6.44
Ferritin (ng/mL)	228.18 (49.14–1233.45)
Total cholesterol (mg/dL)	134.86 ± 26.45
LDL cholesterol (mg/dL)	67.92 ± 20.63
HDL cholesterol (mg/dL)	46.04 ± 13.00
Triglyceride (mg/dL)	104.95 ± 47.23
Calcium (mg/dL)	8.36 ± 0.38
Phosphorus (mg/dL)	4.10 ± 0.66
Intact PTH (mg/dL)	313.23 ± 159.41
C-reactive protein (mg/dL)	0.42 ± 0.43
Kt/Vurea	1.53 ± 0.21
Body mass index (kg/m^2^)	23.58 ± 3.02
Lean tissue index (kg/m^2^)	12.71 ± 3.17
Fat tissue index (kg/m^2^)	9.80 ± 3.59
Body cell mass index (kg/m^2^)	6.92 ± 2.25
PhA (°)	4.28 ± 0.94
Darbepoetin dosage (µg/week)	25.08 ± 13.41

HDL: high-density lipoprotein; LDL: low-density lipoprotein; OH: overhydration/extracellular water ratio; PTH: parathyroid hormone; TIBC: total iron-binding capacity; PhA: phase angle. Continuous variables are expressed as mean ± standard deviation, while categorical variables are expressed as number (percentage).

### Comparisons of variables by Hb-CV level

The analysis that divided the median Hb-CV value no difference in age, sex, presence of diabetes, or HD duration. In addition, Hb-Avg and biochemical and nutritional parameters did not differ between the two groups. Finally, there was no intergroup difference with respect to darbepoetin dose received (Table 2).

**Table 2.** Comparisons of variables by Hb-CV in HD patients (*N* = 76).

	Hb-CV ≤ 9.47 (*n* = 38)	Hb-CV > 9.47 (*n* = 38)	*p* value
Age (years)	65.40 ± 11.94	66.29 ± 12.03	0.746
Sex (*n*, %)			0.491
Male	21 (55.3)	18 (47.4)	
Female	17 (44.7)	20 (52.6)	
Dialysis duration (months)	76.55 ± 55.71	68.16 ± 46.29	0.477
Diabetes mellitus (*n*, %)	17 (44.7)	23 (60.5)	0.168
Hemoglobin (g/dL)	10.75 ± 0.42	10.85 ± 0.59	0.364
Darbepoetin dosage (µg/week)	23.84 ± 15.12	26.26 ± 11.64	0.441
Albumin (g/dL)	3.82 ± 0.20	3.85 ± 0.24	0.511
Iron (µg/dL)	73.48 ± 18.60	74.09 ± 17.12	0.811
TIBC (µg/dL)	262.70 ± 27.78	251.63 ± 32.96	0.118
Transferrin saturation (%)	27.94 ± 6.08	29.67 ± 6.75	0.243
Ferritin (ng/mL)	183.93 ± 122.86	272.44 ± 248.15	0.053
Total cholesterol (mg/dL)	137.55 ± 23.82	132.20 ± 28.90	0.381
LDL cholesterol (mg/dL)	70.93 ± 18.25	67.93 ± 22.55	0.206
HDL cholesterol (mg/dL)	45.65 ± 13.41	46.35 ± 12.70	0.817
Triglyceride (mg/dL)	104.64 ± 45.46	105.15 ± 49.49	0.963
Calcium (mg/dL)	8.30 ± 0.31	8.41 ± 0.43	0.200
Phosphorus (mg/dL)	4.17 ± 0.64	4.02 ± 0.67	0.297
PTH (mg/dL)	312.43 ± 162.62	313.61 ± 158.31	0.975
C-reactive protein (mg/dL)	0.47 ± 0.48	0.37 ± 0.36	0.339
Body mass index (kg/m^2^)	23.84 ± 3.37	23.32 ± 2.65	0.456
Lean tissue index (kg/m^2^)	12.93 ± 3.74	12.50 ± 2.51	0.557
Fat tissue index (kg/m^2^)	9.88 ± 4.09	9.73 ± 3.06	0.857
Body cell mass index (kg/m^2^)	7.05 ± 2.66	6.79 ± 1.79	0.618
PhA (°)	4.27 ± 0.96	4.28 ± 0.94	0.941

CV: coefficient of variation; Hb-CV: hemoglobin coefficient of variation; HD: hemodialysis; HDL: high-density lipoprotein; LDL: low-density lipoprotein; PTH: parathyroid hormone; TIBC: total iron-binding capacity; PhA: phase angle. Continuous variables are expressed as mean ± standard deviation or median (range), while categorical variables are expressed as number (percentage).

### Comparisons of variables by Hb-Med level

The analysis that divided the median Hb value found that compared to patients with an Hb-Med ≤ 10.77 group, those with an Hb-Med >10.77 had a significantly higher proportion of male sex and significantly higher serum albumin level, TIBC, and PhA (65.8 versus 34.2%, 3.91 ± 0.21 versus 3.76 ± 0.21 mg/dL, 264.93 ± 33.41 versus 249.41 ± 26.10 µg/dL, and 4.49 ± 0.92 versus 4.06 ± 0.93°, respectively; *p* < 0.05). On the contrary, the darbepoetin dose received in the Hb-Med >10.77 group was lower than that in the Hb-Med ≤ 10.77 group (18.02 ± 9.50 versus 32.15 ± 13.10; *p* < 0.05; Table 3).

**Table 3.** Comparison of variables by Hb-Med level in HD patients (*N* = 76).

	Hb-Med ≤ 10.77 (*n* = 38)	Hb-Med > 10.77 (*n* = 38)	*p* value
Age (years)	65.29 ± 12.67	66.39 ± 11.26	0.689
Sex			0.012*
Male (*n*, %)	14 (36.8)	25 (65.8)	
Female (*n*, %)	24 (63.2)	13 (34.2)	
Dialysis duration (months)	74.63 ± 44.64	70.08 ± 57.27	0.700
Diabetes mellitus (*n*, %)	20 (50.0)	20 (50.0)	1.000
Hemoglobin (g/dL)	**10.45 ± 0.23**	**11.15 ± 0.48**	<0.001*
Darbepoetin dosage (µg/week)	**32.15 ± 13.10**	**18.02 ± 9.50**	<0.001*
Albumin (g/dL)	**3.76 ± 0.21**	**3.91 ± 0.21**	0.003*
Iron (µg/dL)	71.04 ± 18.86	76.53 ± 16.37	0.180
TIBC (µg/dL)	**249.41 ± 26.10**	**264.93 ± 33.41**	0.027*
Transferrin saturation (%)	28.38 ± 6.24	29.24 ± 6.70	0.563
Ferritin (ng/mL)	255.70 ± 223.35	200.67 ± 171.04	0.232
Total cholesterol (mg/dL)	131.44 ± 27.96	138.31 ± 24.74	0.260
LDL cholesterol (mg/dL)	65.49 ± 20.58	70.38 ± 20.59	0.304
HDL cholesterol (mg/dL)	45.49 ± 12.68	46.51 ± 13.42	0.734
Triglyceride (mg/dL)	101.47 ± 47.16	108.32 ± 47.63	0.531
Calcium (mg/dL)	8.29 ± 0.38	8.42 ± 0.38	0.146
Phosphorus (mg/dL)	4.10 ± 0.69	4.09 ± 0.63	0.965
PTH (mg/dL)	323.41 ± 165.59	302.63 ± 15.49	0.573
C-reactive protein (mg/dL)	0.47 ± 0.45	0.36 ± 0.41	0.271
Body mass index (kg/m^2^)	23.07 ± 3.02	24.08 ± 2.98	0.146
Lean tissue index (kg/m^2^)	12.01 ± 3.32	13.42 ± 2.89	0.051
Fat tissue index (kg/m^2^)	9.93 ± 3.63	9.67 ± 3.59	0.754
Body cell mass index (kg/m^2^)	6.42 ± 2.36	7.42 ± 2.05	0.053
PhA (°)	**4.06 ± 0.93**	**4.49 ± 0.92**	0.043*

Med: median value; HDL: high-density lipoprotein; LDL: low-density lipoprotein; PTH: parathyroid hormone; TIBC: total iron-binding capacity; PhA: phase angle. Continuous variables are expressed as mean ± standard deviation, while categorical variables are expressed as number (percentage). *p < 0.05.

The bold values indicate statistical difference (*p*<0.05) between the two groups.

### Correlations between clinical and biochemical variables and PhA

**Figure 2.**
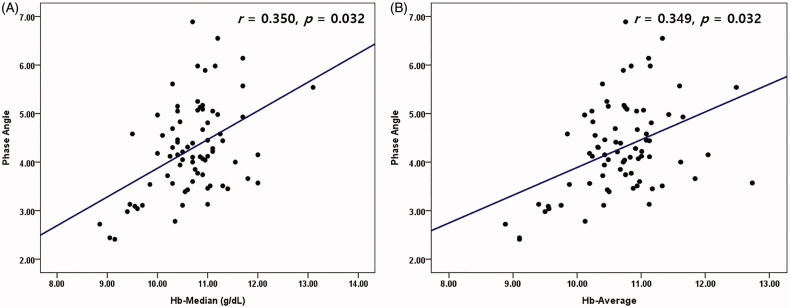
(A,B) Correlations among Hb-median, Hb-average, and phase angle in HD patients (*N* = 76).

Age, female sex, darbepoetin dosage, and OH were negatively correlated with PhA (*r* = −0.490, −0.345, −0.287, and −0.558 respectively; *p* < 0.05). Serum albumin, phosphorus, TIBC, Hb-Med and Hb-Avg were positively correlated with PhA (*r* = 0.457, 0.338, 0.329, 0.350 and 0.349; *p* < 0.05) (Table 4 and Figure 2).

**Table 4.** Correlations between clinical, biochemical variables and PhA (*n* = 76).

Variable	PhA
Age (years)	–0.490 (*p* < 0.001)*
Sex (male : female)	–0.345 (*p* = 0.002)*
Diabetes mellitus	**–**0.104 (*p* = 0.370)
Darbepoetin dosage	–0.287 (*p* = 0.013)*
Iron	0.176 (*p* = 0.128)
TIBC	0.329 (*p* = 0.027)*
Transferrin saturation	0.069 (*p* = 0.551)
Ferritin	**–**0.063 (*p* = 0.589)
Albumin	0.457 (*p* < 0.001)*
Calcium	0.033 (*p* = 0.778)
Phosphorus	0.338 (*p* = 0.003)*
PTH	−0.006 (*p* = 0.956)
C-reactive protein	**–**0.212 (*p* = 0.066)
Total cholesterol	0.030 (*p* = 0.798)
LDL cholesterol	0.016 (*p* = 0.894)
HDL cholesterol	**–**0.004 (*p* = 0.973)
Triglyceride	0.155 (*p* = 0.182)
Dialysis vintage	**–**0.105 (*p* = 0.366)
Hb-Avg	0.349 (*p* = 0.032)*
Hb-Min	0.039 (*p* = 0.760)
Hb-Max	0.091 (*p* = 0.433)
Hb-RAN	0.147 (*p* = 0.188)
Hb-SD	0.168 (*p* = 0.162)
Hb-Med	0.350 (*p* = 0.032)*
Hb-CV	0.221 (*p* = 0.125)
OH	–0.558 (*p* = 0.001)*

PhA: phase angle; Hb: hemoglobin; Hb-Avg: average of Hb; Hb-CV: coefficient of variation of Hb; Hb-Max: maximum of Hb; Hb-Med: median of Hb; Hb-Min: minimum of Hb; Hb-RAN: range of Hb; Hb-SD: standard deviation of Hb; HDL: high-density lipoprotein; LDL: low-density lipoprotein; OH: overhydration/extracellular water ratio; PTH: parathyroid hormone; TIBC: total iron-binding capacity. Spearman’s (non-parametric) correlation was used to test for associations between clinical and biochemical variables and PhA.*p < 0.05.

The bold values indicate statistical difference (*p*<0.05) between correlation.

### Multivariate linear regression analysis with PhA as dependent variable

In multiple linear regression analysis, PhA was positively associated with Hb-Med, and serum albumin level (*β* = 0.085 and 1.350, respectively; *p <* 0.05), whereas PhA was negatively associated with age and female sex (*β* = −0.041 and −0.477, respectively; *p* < 0.05; Table 5).

**Table 5.** Multivariate linear regression analysis with PhA as a dependent variable in HD patients (*N* = 76).

	PhA		
	β	SE	*p* value
Age	−0.041	0.011	0.001 *
Female	−0.477	0.243	0.048 *
Albumin	1.350	0.570	0.024 *
Hb-Med	0.085	0.522	0.045 *

Selected variables: age, sex, serum albumin level, phosphorus level, TIBC, Hb-CV, Hb-SD, Hb-RAN, Hb-Min, Hb-Max, Hb-Med, OH, and darbepoetin dosage. PhA: phase angle; Hb-Avg: average of Hb; Hb-CV: coefficient of variation of Hb; Hb-Max: maximum of Hb; Hb-Med: median of Hb; Hb-Min: minimum of Hb; Hb-RAN: range of Hb; Hb-SD: standard deviation of Hb; HD: hemodialysis; TIBC: total iron-binding capacity; OH: overhydration/extracellular water ratio. *p < 0.05.

### ROC curves of Hb-Med, Hb-CV for predicting PhA > 5.00°

**Figure 3.**
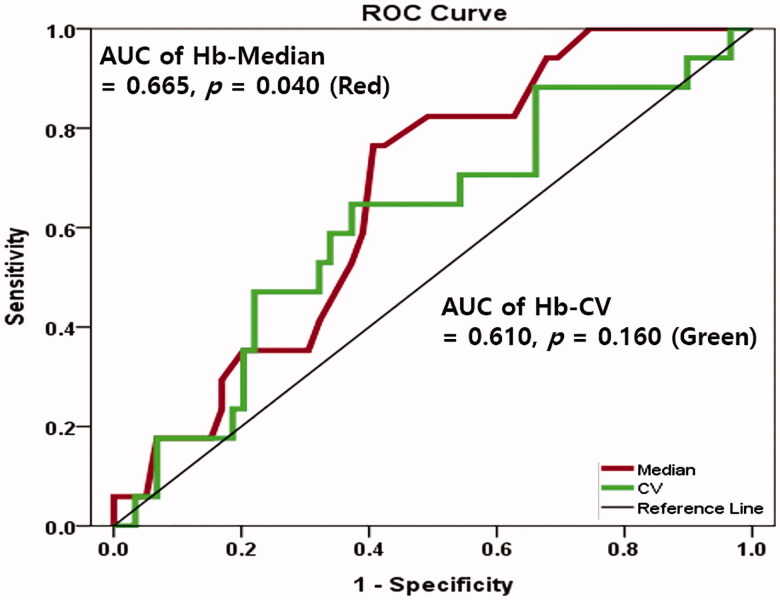
Receiver operating characteristics (ROC) curves of Hb-median (red) and Hb-CV (green) for predicting PA > 5.00°.

The AUC of Hb-Med was 0.665 (95% confidence interval 0.535–0.794, *p* = 0.040) in predicting PhA > 5.00°. Optimal Hb-Med cutoff value was 10.77 g/dL (sensitivity 76.5%, specificity 59.3%). On the contrary, the AUC of Hb-CV was 0.610 (95% confidence interval 0.457–0.764, *p* = 0.168) in predicting PhA > 5.00° (Figure 3).

## Discussion

We evaluated the cross-sectional correlation between Hb variability and difference in nutritional status using BCM in chronic HD patients over the last 24 months. The main findings of our study were that, unexpectedly, there was no association between Hb variability using Hb-CV, Hb-SD, Hb-RAN, and all nutritional parameters including PhA. On the contrary, only PhA among several nutritional parameters was associated with Hb-Med and Hb-Avg. Optimal Hb-Med cutoff value to predict the well-nourished status in chronic HD patients was 10.77 g/dL. Finally, the HD patients with a higher Hb-Med showed lower average weekly darbepoetin dosage, and darbepoetin dosage were negatively correlated with PhA.

Hb variability in HD patients was first described by Lacson and Berns in 2003, and most studies have investigated the relationship between Hb variability and overall mortality rate [10–12]. In a recent retrospective study including 252 patients, Hb variability was independently associated with the cardiovascular mortality of HD patients, and patients in the highest Hb variation group had a nine times higher cardiovascular death risk than those in the lowest Hb variation group [4]. In a meta-analysis, the investigators analyzed the relationship between Hb variability and all-cause mortality and found a 9% increase in the adjusted rate of death for each 1 g/dL increase in Hb variability [5]. In contrast, few studies have examined the relationship between Hb variability and nutritional status. A recent study of 754 HD patients by Rattanasompattikul et al., with nutritional markers such as MIS, considered wasting an independent factor for ESA resistance. They found that low levels of nutritional markers were independent predictors of a decreased response to ESAs [13]. In another observational multicenter study, the investigators found that in HD patients, those with a high BMI (≥30 kg/m^2^) required a lower ESA dose and had a better response to anemia treatment [14]. These studies focused on the relationship between ESA responsiveness and nutritional status.

Among available indicators of nutritional status, PhA measured by BIA or BCM is promising. Several recent papers have reported that PhA is a good indicator of nutritional status and useful predictor of mortality [15–18]. PhA can be used to screen individuals prone to mortality. In particular, BIA-provided PhA is the most potent predictor of malnutrition and associated with death in patients with CKD [19,20]. 5.00° is usually considered as a cutoff PhA value for estimating well-nourished status proposed in previous research [6]. One paper explored the relationship between PhA and ESA responsiveness. Colin et al. found that patients with a lower PhA had a lower Hb and hematocrit. In addition, they suggested that patients with a PhA > 5.00° responded satisfactorily to treatment with ESA, while those with a lower PhA did not respond to treatment [21]. However, no recent studies have investigated the relationship between PhA and Hb variability. In our study, there was no association between Hb variability on Hb-CV, Hb-SD, Hb-RAN, or any nutritional parameters including PhA. On the contrary, only PhA among other nutritional parameters was significantly associated with Hb-Med and Hb-Avg. The reason for this unexpected finding resulted from the low-amplitude Hb-variability in our study population. The median value of Hb-CV in our study was only 9.47, which indicates statistically low-amplitude Hb variability. In Korea, the target Hb level was 10–11 g/dL according to reimbursement regulations of the Korea Health Insurance Review & Assessment Service. Strict dose adjustment of darbepoetin was performed according to monthly Hb level in every HD center. The Hb level in our study was controlled within a narrow range. This was the reason why our patients could not be classified into six groups: consistently low, consistently within the target range, consistently high, low-amplitude fluctuation with low Hb levels, low-amplitude fluctuation with high Hb levels, and high-amplitude fluctuation, the main applied methods in a previous study of Hb variability. Our study was forced on investigating the effects of Hb variability using Hb indexes such as Hb-CV, Hb-SD, and Hb-RAN. Taken together, the factors that related the PhA of HD patients are the maintenance of appropriate Hb-Med and Hb-Avg values for a period of time, especially in the situation of low-amplitude Hb variability. In our study, well-known nutritional markers such as albumin level, phosphorus level, and TIBC were positively correlated with PhA, whereas older age and female sex were negatively correlated with PhA. Considering these results, the reproducibility of our results is guaranteed.

Another major finding of our study was that HD patients with a higher Hb-Med showed lower average weekly darbepoetin dosages and that darbepoetin dosage was negatively correlated with PhA. There have been conflicting results between ESA responsiveness, Hb, and PhA. In a previous study, Han et al. demonstrated that PhA was positively associated with a geriatric nutritional risk index, lean tissue index, and albumin level in non-dialytic stage 5 CKD and PD patients. However, there was no association between PhA and Hb level in those patients [22]. González et al. reported that an MIS ≤ 6 indicated a possible response to treatment with ESA but that a PhA ≥ 5.00° showed a trend that was not statistically significant [23]. On the contrary, Shin et al. demonstrated that patients with a PhA ≥ 4.50° received lower weekly doses of ESAs and monthly doses of intravenous iron than those with a PhA < 4.50°. They concluded that HD patients with a low PhA have poor responsiveness to anemia management including ESAs and intravenous iron [18]. The previous two studies had a short follow-up period of 3 months, while one study was conducted in non-dialytic CKD stage 5 and peritoneal dialysis patients, not in HD patients. However, our study followed HD patients for 24 months; therefore, we found that the lower the PhA, the higher the required ESA dose.

The results of our study should be interpreted with caution given the following limitations. First, this was a cross-sectional and single-center study, which could have resulted in information bias, and included a relatively small sample size. However, we followed up with regular medical records for 2 years; thus, missing or incorrect information was likely minimized. Second, the iron administration would have influenced the Hb control. However, the dose of iron administered to the patient could not be determined, although TIBC and ferritin levels were not different and would not have a significant effect in this study.

## Conclusions

Phase angle values, a good indicator of nutritional status, are associated with median value of Hb rather than Hb variability in chronic HD patients. PhA provides practical information to predict responsiveness to ESAs in the same population.
